# Supplementary material for: Retinol-Binding Protein 4 and Visfatin Levels in Patients with Periodontitis and Obesity/Overweight: A Systematic Review and Meta-Analysis
Source: Curr Issues Mol Biol. 2023 Dec 7;45(12):9838–50. doi: 10.3390/cimb45120614 (PMC10742403; doi:10.3390/cimb45120614)
Supplement: Supplementary file 1 [file cimb-45-00614-s001.zip › cimb-2730568-supplementary.pdf]

Supplementary file

Retinol-Binding Protein 4 and Visfatin Levels in Patients with Periodontitis and Obesity/Overweight: A Systematic Review and Meta-Analysis

Yuwei Zhang [1,2,†], Ru Jia [1,2,†], Yifei Zhang [1,2], Rui Zou [1,2], Lin Niu [1,2,3,*] and Shaojie Dong [1,2,3,*]

1. Key Laboratory of Shaanxi Province for Craniofacial Precision Medicine Research, College of Stomatology, Xi'an Jiaotong University, Xi'an 710004, Shaanxi Province, China
2. Clinical Research Center of Shaanxi Province for Dental and Maxillofacial Diseases, Xi'an 710004, Shaanxi Province, China
3. Department of Prosthodontics, College of Stomatology, Xi'an Jiaotong University, Xi'an 710004, Shaanxi Province, China

# These authors contributed equally to this work.

* Corresponding authors.

Lin Niu, niulin@xjtu.edu.cn; Shaojie Dong, dongshaojie@xjtu.edu.cn.

Table S1 – Inclusion and exclusion criteria and studies excluded after full-text analysis and related reasons

Inclusion criteria	Exclusion criteria
Randomized clinical trials;<br>Controlled clinical trials;<br>Reported the given cytokines/ adipocytokines levels;<br>Published in English;<br>Suitable obesity & periodontitis criterion: (Obesity: recommended by WHO; Periodontitis: widely accepted & preferred 2017 world workshop [1])	Laboratory animal science;<br>Case-control studies;<br>Cross-sectional;<br>Case series;<br>Systematic reviews;<br>Literature reviews;<br>Data published as conference abstracts;<br>Studies without certain MD or CI data;<br>Clinical analysis involving periodontitis individuals with systemic conditions other than obesity

Study	Reason for exclusion

Raghavendra NM, Pradeep AR, Kathariya R, Sharma A, Rao NS, Naik SB. Effect of non surgical periodontal therapy on gingival crevicular fluid and serum visfatin concentration in periodontal health and disease. *Dis Markers*. 2012;32(6):383-388. doi:10.3233/DMA-2012-0897	The study did not include individuals with obesity.
Rajasekar A. Correlation of salivary visfatin levels in obese and NON-OBESE population with periodontal status. *J Oral Biol Craniofac Res*. 2023;13(1):67-70. doi:10.1016/j.jobcr.2022.11.004	In this study, due to the classification, the specific statistics of visfatin levels of patients with periodontitis could not be retrieved.
Begum, S., B. Pratebha, J. Muthu, V. Priyadharshini, K. Vineela, and R. Saravanakumar, Correlation of Salivary Retinol Binding Protein 4 (RBP4) Levels and Periodontal Severity in Patients with Chronic Periodontitis. *International Journal of Life Science and Pharma Research*, 2022. 12: p. L128-L135. doi:10.22376/ijpbs/lpr.2022.12.6.SP25.L128-135	The study did not include individuals with obesity.
Martinez-Herrera M, López-Domènech S, Silvestre FJ, et al. Chronic periodontitis impairs polymorphonuclear leucocyte-endothelium cell interactions and oxidative stress in humans. *J Clin Periodontol*. 2018;45(12):1429-1439. doi:10.1111/jcpe.13027	In this study, the specific statistics of RBP4 levels could not be retrieved.
Silva-Boghossian CM, Dezonne RS. What Are the Clinical and Systemic Results of Periodontitis Treatment in Obese Individuals?. *Curr Oral Health Rep*. 2021;8(3):48-65. doi:10.1007/s40496-021-00295-5	This article was a review.
Virto L, Cano P, Jiménez-Ortega V, et al. Obesity and periodontitis: An experimental study to evaluate periodontal and systemic effects of comorbidity. *J Periodontol*. 2018;89(2):176-185. doi:10.1902/jop.2017.170355	This article was an animal experimental study.
Deschner J, Eick S, Damanaki A, Nokhbehsaim M. The role of adipokines in periodontal infection and healing. *Mol Oral Microbiol*. 2014;29(6):258-269. doi:10.1111/omi.12070	This article was a review.
Rezaei M, Bayani M, Tasorian B, Mahdian S. The comparison of visfatin levels of gingival crevicular fluid in systemic lupus erythematosus and chronic periodontitis patients with healthy subjects. *Clin Rheumatol*. 2019;38(11):3139-3143. doi:10.1007/s10067-019-04708-w	The study did not include individuals with obesity.
Nogueira AV, Nokhbehsaim M, Eick S, et al. Regulation of visfatin by microbial and biomechanical signals in PDL cells. *Clin Oral Investig*. 2014;18(1):171-178. doi:10.1007/s00784-013-0935-1	This article was an *in vitro* experimental study.
Memmert S, Damanaki A, Nogueira AVB, et al. Regulation of tyrosine hydroxylase in periodontal fibroblasts and tissues by obesity-associated stimuli. *Cell Tissue Res*. 2019;375(3):619-628.	This article was an experimental study.

doi:10.1007/s00441-018-2941-8	

Damanaki A, Memmert S, Nokhbehsaim M, et al. Impact of obesity and aging on crestal alveolar bone height in mice. *Ann Anat*. 2018;218:227-235. doi:10.1016/j.aanat.2018.04.005	This article was an animal experimental study.
Nokhbehsaim M, Eick S, Nogueira AV, et al. Stimulation of MMP-1 and CCL2 by NAMPT in PDL cells. *Mediators Inflamm*. 2013;2013:437123. doi:10.1155/2013/437123	This article was an *in vitro* experimental study.
Memmert S, Damanaki A, Nokhbehsaim M, et al. Regulation of somatostatin receptor 2 by proinflammatory, microbial and obesity-related signals in periodontal cells and tissues. *Head Face Med*. 2019;15(1):2. Published 2019 Jan 4. doi:10.1186/s13005-018-0185-1	This article was an experimental study.
Nokhbehsaim M, Keser S, Jäger A, Jepsen S, Deschner J. Regulation of regenerative periodontal healing by NAMPT. *Mediators Inflamm*. 2013;2013:202530. doi:10.1155/2013/202530	This article was an *in vitro* experimental study.

Table S2 - Risk of bias assessment of included studies according to the ROBINS-I tool.

	ÇETİNER et al., 2018	Martinez-Herrera et al., 2017
Domain 1: Confounding factors	Low	Low
Domain 2: Selection of participants	Moderate	Moderate
Domain 3: Intervention classification	Low	Low
Domain 4: Deviation from intervention	Low	Low
Domain 5: Missing data	Low	Moderate

	Domain 6: Measurement of outcome	Low	Low
	Domain 7: Selection of reported result	Moderate	Low
	ROBINS-I overall score	Low	Low

† ROBINS-I is a tool which assesses the risk of bias in non-randomized studies of interventions by looking into pre-intervention, intervention and post-intervention domains. Studies with low risk of bias (no or one moderate concern in the included domains) are comparable to randomized controlled trials. Studies with moderate risk of bias (up to four moderate concerns in the included domains) can be characterized as credible but cannot considered comparable to a well performed randomized trial. Studies with serious risk of bias (at least one serious concern or multiple moderate concerns in the included domains) have important problems in the design. Studies with critical risk of bias (critical concerns or multiple serious concerns in the included domains) are too problematic to provide useful evidence on the intervention effect.

Table S3- Quality assessment of incorporated studies using the Newcastle-Ottawa Scale tool.

Study	Selection	Comparability	Exposure	Score	Quality
Matern et al., 2020	☆☆☆	☆	☆☆☆	7	Good
Kanoriya et al., 2016	☆☆☆☆	☆	☆☆☆	8	Good
Jia et al., 2023	☆☆☆	☆	☆☆☆	7	Good
Li et al., 2018	☆☆☆	☆	☆☆☆	7	Good
Doğan et al., 2022	☆☆☆	☆	☆☆☆	7	Good

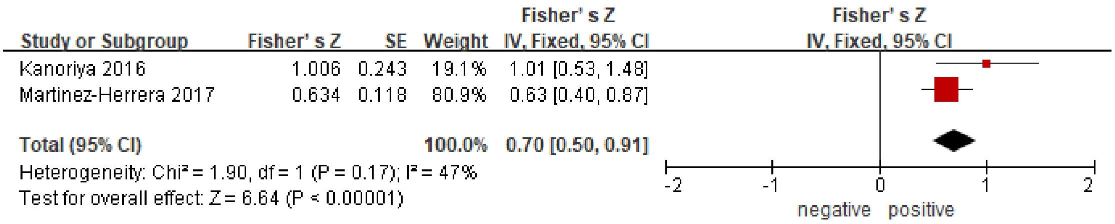

Figure S1. Subgroup analysis of the association of serum RBP4 levels with body mass index (BMI) in patients with obesity and periodontitis (OP). The fixed effect model and Fisher's Z with 95% CI were applied.

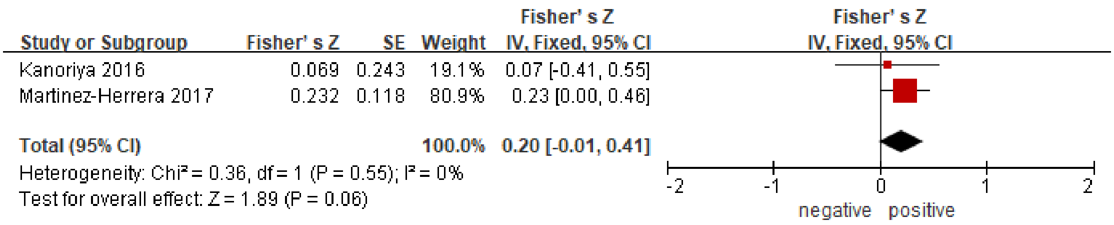

Figure S2. Subgroup analysis of the association of serum RBP4 levels with probing depth (PD) in patients with obesity and periodontitis (OP). The fixed effect model and Fisher's Z with 95% CI were applied.

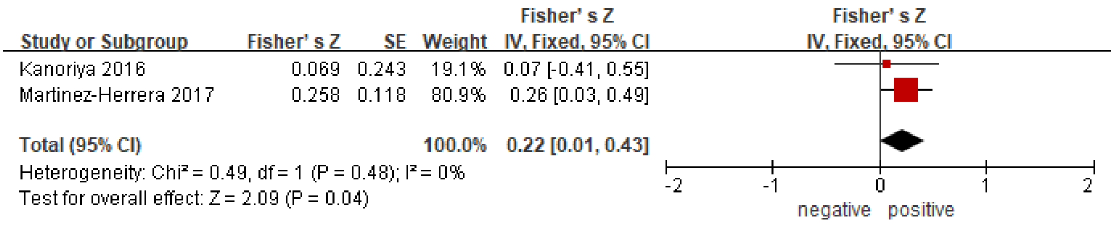

Figure S3. Subgroup analysis of the association of serum RBP4 levels with clinical attachment loss (CAL) in patients with obesity and periodontitis (OP). The fixed effect model and Fisher's Z with 95% CI were applied.

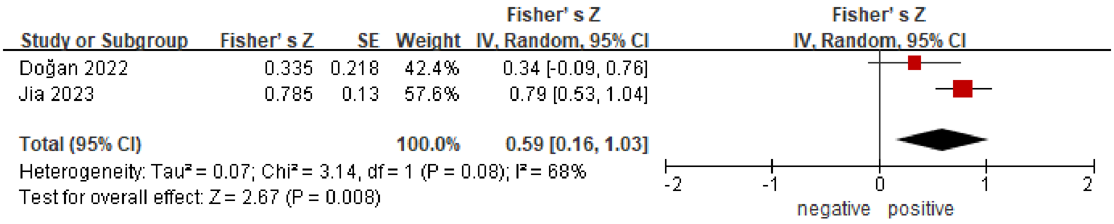

Figure S4. Subgroup analysis of the association of serum visfatin levels with probing depth (PD) in patients with obesity and periodontitis (OP). The random effect model and Fisher's Z with 95% CI were applied.

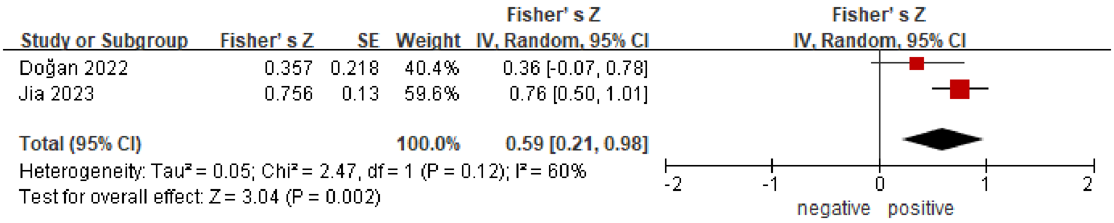

Figure S5. Subgroup analysis of the association of serum visfatin levels with clinical attachment loss (CAL) in patients with obesity and periodontitis (OP). The random effect model and Fisher's Z with 95% CI were applied.

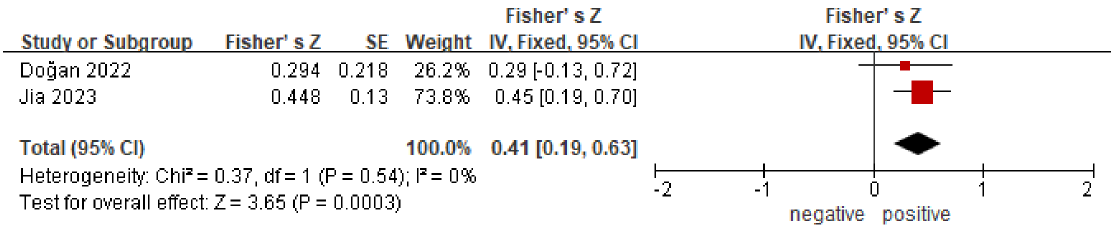

Figure S6. Subgroup analysis of the association of serum visfatin levels with visible periodontal plaque index (PI)

in patients with obesity and periodontitis (OP). The fixed effect model and Fisher's Z with 95% CI were applied.

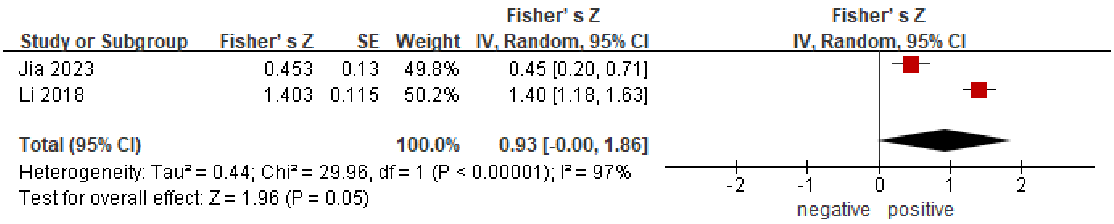

Figure S7. Subgroup analysis of the association of serum visfatin levels with total cholesterol (TC) levels in patients with obesity and periodontitis (OP). The random effect model and Fisher's Z with 95% CI were applied.

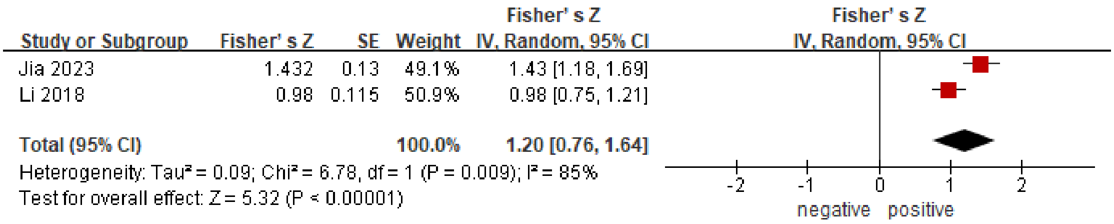

Figure S8. Subgroup analysis of the association of serum visfatin levels with triglycerides (TG) levels in patients with obesity and periodontitis (OP). The random effect model and Fisher's Z with 95% CI were applied.

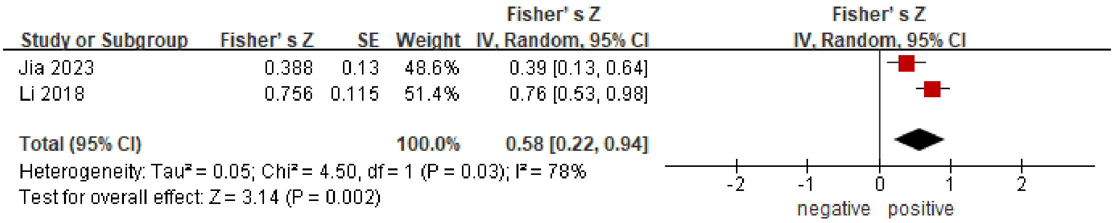

Figure S9. Subgroup analysis of the association of serum visfatin levels with fasting blood glucose (FBG) levels in patients with obesity and periodontitis (OP). The random effect model and Fisher's Z with 95% CI were applied.

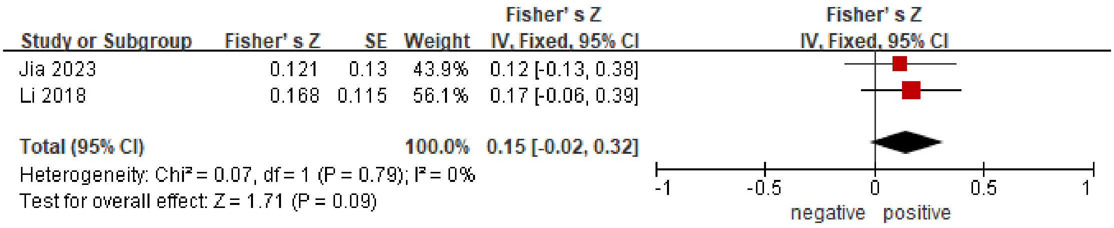

Figure S10. Subgroup analysis of the association of serum visfatin levels with glycosylated hemoglobin (HbA1c） levels in patients with obesity and periodontitis (OP). The fixed effect model and Fisher's Z with 95% CI were applied.

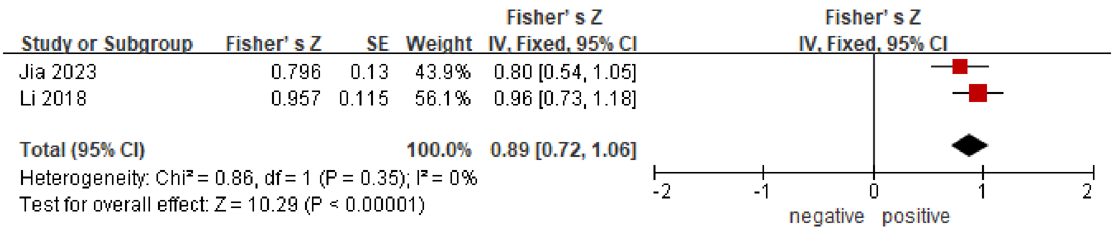

Figure S11. Subgroup analysis of the association of serum visfatin levels with low density lipoprotein cholesterol (LDL-C) levels in patients with obesity and periodontitis (OP). The fixed effect model and Fisher's Z with 95% CI were applied.

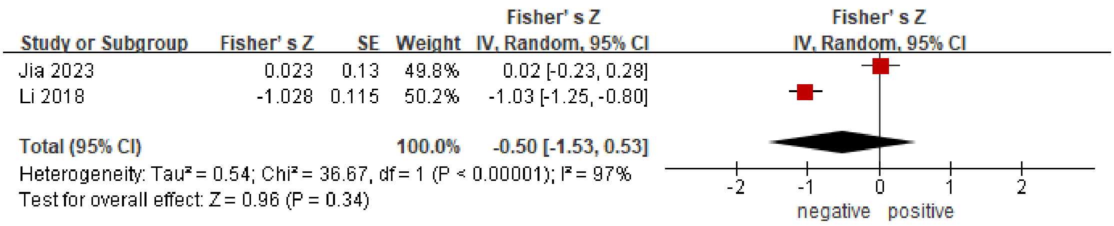

Figure S12. Subgroup analysis of the association of serum visfatin levels with high density lipoprotein cholesterol (HDL-C) levels in patients with obesity and periodontitis (OP). The fixed effect model and Fisher's Z with 95% CI were applied.

References

1. Papapanou, P.N., M. Sanz, N. Buduneli, T. Dietrich, M. Feres, D.H. Fine, et al., *Periodontitis: Consensus report of workgroup 2 of the 2017 World Workshop on the Classification of Periodontal and Peri-Implant Diseases and Conditions.* J Periodontol, 2018. **89 Suppl 1**: p. S173-s182.
